# Asthma in Rhinosinusitis: A Survey from Iran

**Published:** 2016-07

**Authors:** Mehdi Bakhshaee, Mohamad-Reza Majidi, Vahideh Gharavi, Fatemeh-Sadat Alavizadeh, Rahman Movahed, Parasto Asnaashari, Amir-Mohammad-Hashem Asnaashari

**Affiliations:** 1 *Sinus and Surgical Endoscopic Research Center, Faculty of Medicine, Mashhad University of Medical Sciences, Mashhad, Iran. *; 2 *Department of Otorhinolaryngology, Mashhad University of Medical Sciences, Mashhad, Iran.*; 3 *COPD Research Center, Faculty of Medicine, Mashhad University of Medical Sciences, Mashhad, Iran.*

**Keywords:** Asthma, Sinusitis, Spirometry

## Abstract

**Introduction:**

The coexistence of asthma and chronic rhinosinusitis (CRS) is more common than expected given their individual prevalence in the general population and may affect patient’s quality of life. The aim of this study was to evaluate the prevalence of asthma in chronic rhinosinusitis in Mashhad, Northeast Iran.

**Materials and Methods:**

This study was performed in two university hospital from November 2012 for 12 months. In total, 153 patients with chronic rhinosinusitis were enrolled and referred to a particular pulmonologist for asthma evaluation.

**Results:**

The mean age of participants was 40.54±13.11 years, and 41.8% were male. In total, 63.4% of patients had the polypoid form of CRS. The proportion of patients in this study with asthma was 41.8%, compared with a general asthma prevalence in this region of 13.5%.

**Conclusion:**

There is a high prevalence of asthma among patients with CRS, but it often remains undiagnosed. Asthma in CRS patients should be diagnosed and treated in order to improve patient’s quality of life. We recommend an evaluation of the lower airways in all of these patients as well as further studies in this field.

## Introduction

Chronic rhinosinusitis (CRS), defined by inflammation of the nasal and paranasal sinus mucosa for ≥12 weeks, represents one of the most common healthcare problems worldwide, afflicting approximately 31 million Americans (1). CRS can be divided two subgroups; with and without nasal polyposis. Asthma is a reversible small-airway disease, with a prevalence that has increased in recent decades to become a major cause of disability and death (2). The association and coexistence of asthma and sinusitis has been understood for over 70 years, and the two conditions are believed to be manifestations of a single underlying disease in different parts of the respiratory tract. However, the coexistence of asthma and CRS is more common than would expected given their individual prevalence in the general population, although this association is more common in certain regions of the world than others (3).

This coexistence of CRS with asthma should be diagnosed and treated as soon as possible in order to improve the patient’s quality of life. Therefore, we designed the current study to evaluate the prevalence of asthma in CRS in Mashhad, Northeast Iran.

## Materials and Methods

This cross-sectional study was performed in ear, nose, and throat respiratory clinics of Ghaem and Imam Reza University Hospitals, Mashhad University of Medical Sciences, Mashhad city (Northeast Iran) from November 2012 to November 2013. The proposal was approved by the university 

ethics committee, and written informed consent was provided by each participant.

Patients with CRS diagnosed by radiologic and endoscopic studies based on the European position paper on rhinosinusitis and nasal polyps (EPOS) 2012 criteria were enrolled and then referred to a particular pulmonologist for asthma evaluation. The diagnosis of asthma was confirmed based on clinical symptoms and spirometric data indicative of reversible obstruction of small airways. Obstruction was defined by forced expiratory volume in 1 second (FEV_1_) / forced vital capacity (FVC) <75%, and reversibility was defined as a 12–15% and 200 ml increase in FEV_1_ with the use of two puffs of a short-acting beta-2 agonist (SABA) (4).

## Results

In total, 153 patients with a definite diagnosis of rhinosinusitis were studied from November 2012 for 12 months. The diagnosis of CRS was confirmed by computed tomography (CT) scan and sinus endoscopy. The mean age of patients was 40.54 ±13.11 years; 41.8% were male and 58.2% female.

Among the major clinical symptoms of CRS, nasal airway obstruction (in all cases) and facial pain and/or pressure (in 97.4%) had the highest prevalence (Table.1). In total, 63.4% of patients had the polypoid form of CRS while 36.6% had the non-polypoid form. Among patients with polypoid CRS, 56.9% presented with the severe form of polyposis.

**Table 1:** Frequekncy of presenting symptoms among CRS patients

**Symptoms**	**Frequency (no.)**	**Percentage (%)**	**Symptoms**
Major Symptoms Minor Symptoms	Nasal airway Obstruction	153	100
	Facial pressure/pain	149	97.4
	Purulent discharge	147	96.1
	Discolored PND	133	86.9
	Anosmia	85	55.6
	Hyposmia	52	34.0
	Halitosis	69	45.1
	Headache	64	41.8
	Otalgia	42	27.5
	Malaise	34	22.2
	Dental pain	6	3.9
	Fever	4	2.6

PND: post-nasal drip

FEV_1_/FVC was less than 75% in 47 patients (7%) indicating some degree of small-airway disease, but based on diagnostic criteria only 41.8% of CRS patients were suffering from asthma (Tabale.2) Cough was the most common symptom in asthmatic patients (59%), whereas dyspnea was diagnosed in 22.2%. Surprisingly,13.1% of cases had no complaint regarding clinical symptoms of asthma.

In asthmatic patients, the most common contributing factor was active and passive smoking in 20.9% and 10.5% of cases, respectively. Exercise exacerbated the asthma-related symptoms in 22.2% of patients, whereas allergens were considered responsible in 19.6% of cases. A positive family history of allergy and asthma was observed in 51.9% and 40.9% of the patients, respectively.

**Table 2:** Pulunction measurements in CRS patients

**Measurement**		**Range**	**Mean±SD**
FEV_1_/FVC		71.41±11.98	39–87
FEV_1_		2646.29±926.31	680–4860
MEEF		2.65±1.01	0.63–4.93
**Factor**		**Frequency**	**Percentage**
FEV_1_/FVC	<75% >75%	73	47.7%
		80	52.3%
FEV_1_ change after BD	<12% >12%	29 50	36.7% 63.3%
MEEF	<2 >2	34 83	22.2% 54.2%
MEEF	<60% >60%	31 86	20.3% 56.2%
MEEF change after BD	<12% >12%	26 53	32.5% 67.5%

BD: Bronchodilator

Fisher’s exact test was used to study the association between polypoid and non-polypoid CRS with asthma. As displayed in Table 3, a significant correlation was achieved indicating that asthmatic patients constituted a higher percentage of polypoid CRS cases (P=0.012).

**Table 3:** Asthma prevalence in polypoid vs. non-polypoid patients

	**Asthmatic**	**Non-Asthmatic**	**P-value**
Polypoid Rhinosinusitis	52 (81%)	30 (42%)	0.012
Non-polypoid Rhinosinusitis	12 (19%)	42 (58%)	
Total	64	72	

The association between the severity of polypoid rhinosinusitis and asthma was studied using the Kruskal-Wallis test, which showed a meaningful relationship (P<0.05). This indicates that patients with asthma have more severe degrees of rhinosinusitis. One-way analysis of variance (ANOVA) showed a significant correlation between FEV_1_/FVC and the severity of polypoid CRS, indicating mean FEV_1_/FVC decreased with increasing severity of polypoid CRS (P<0.05). Using the same statistical test, a meaningful reverse relationship was found between both the level and percentage of maximum mid-expiratory flow rate (MMEF) and the severity of polypoid CRS (P=0.012 and P<0.05, respectively).

A skin prick test was performed in 102 patients. Based on the Chi-square test, there was no significant association between this test result and the type of CRS (P=0.56). Positive prick test results had an almost similar rate in both the polypoid and non-polypoid CRS groups, while salsola was the most common allergen with a prevalence of 35.6% and 49.1% in the two groups, respectively.

A history of gastroesophageal reflux (GERD) and smoking was noted in 46.1% and 20.9% of patients respectively. According to the Chi-square statistical test, there was no relationship between these two factors in any type of CRS, but there was significant relationship between these two factors and asthma diagnosis in CRS patients.

## Discussion

Nowadays, asthma is one of the most common health problems worldwide. Its prevalence has increased dramatically since 1960 and is today counted as one of the main causes of disability, high medical expenses, and preventable deaths (5). Physicians who manage patients with CRS should also consider a possible diagnosis of asthma in order to provide the best referral protocol and to treat such patients promptly. Such an approach may result a better quality of life for these patients (6).

The prevalence of asthma varies from region to region. Several studies on the prevalence of asthma in Iran have been published, most of them conducted in children. Based on these studies, the prevalence of asthma is approximately 13.5% in the general population (7).

In our study the prevalence of asthma was 41.8% in CRS patients, which is far higher than in the general population. These results are consistent with the study of Staikūniene in Lithuania with a reported asthma prevalence of 39.6% (8). However, in other similar studies asthma prevalence ranged from 2–24% in CRS patients (9-12). In the Western population, asthma occurs in 20–31.9% of CRS patients. However, these figures may differ in developing countries. The prevalence of asthma among CRS cases has been reported as 2–3% in China (10). This could be due to the different immunopathologic characteristics of asthma in CRS cases, mainly due to the lower rate of eosinophilic inflammation in the Chinese population. In general, environmental factors probably have a major role in the prevalence of asthma around the world (13).

Among our studied population, asthmatic patients constituted a greater percentage of polypoid CRS cases. Eighty-eight percent of the asthmatic patients were of the polypoid type, whereas 12% were non-polypoid; showing a meaningful association between asthma and polypoid rhinosinusitis. This is a similar finding as reported in other studies (11,12,14). In contrast, Baraniuk showed that the prevalence of asthma was similar in the polypoid and non-polypoid groups; however it had a greater severity in the polypoid group whereas the non-polypoid group had a higher bronchial hyper-responsiveness rate (15).

In our study, in patients with polypoid CRS, 89% had severe polyposis whereas 7% and 4% had moderate and mild polyposis, respectively. A significant correlation was found between the severity of polypoid CRS and asthma. In general, most studies had similar findings, reporting a direct correlation between the degree of CRS and asthma severity(7,16).However, inconsistent results have also been reported (15,17).

Regarding CRS symptoms, chronic cough, halitosis, and headache had the highest prevalence. Watelet reported that when cough presents, there was an increased risk of concomitant asthma and polypoid CRS (18). In our study, the most common symptom among asthmatics was cough, but the relationship was not statistically significant.

In contrast with the study by Seybt et al. (12), the prevalence of headache in our study was significantly higher among asthmatic patients (65.6% vs. 25%). Wilkinson (19) showed the same association and reported that asthma is an independent risk factor for recurrent headaches. Of note, the relationship between headache and asthma is related to airway hyper-responsiveness and is independent of its atopic nature.

Prick test results were available for only 102 patients, of whom 71.5% had a positive test result. No meaningful correlation was observed between positive prick test results and the type of CRS or asthma. Other studies have reported inconsistent results in this respect (20-23,27,28).

In the current study, we also found a significant relationship between FEV_1_/FVC and degree of polypoid CRS; mean FEV_1_/FVC level decreased with increasing severity of polyposis. Moreover, a meaningful relationship between MMEF and the severity of polypoid CRS was observed, meaning that MMEF decreases with increasing severity of rhinosinusitis. The level and percentage of MMEF varied between the polypoid and non-polypoid groups, showing a greater decrease among polypoid CRS patients. Our findings are consistent with those of Staikūniene et al. and Okayama et al. (8,24).

Among our studied population, 34% complained of hyposmia whereas 55.6% suffered from anosmia; olfactory dysfunction was significantly associated with the type of CRS and the presence of asthma. These findings were in accordance with other similar studies. In addition to considering olfactory dysfunction as one of the characteristics of polypoid CRS, the association between asthma and olfactory dysfunction is also noted (20,11,25).

## Conclusion

Our study demonstrated a high prevalence of asthma among CRS patients. Unfortunately, however, asthma often remains undiagnosed in these patients. Being aware of this correlation may result in the early diagnosis of asthma and a better quality of life for patients; therefore evaluation of lower airways in all CRS patients may be necessary. We suggest further studies in this field.
